# Twelve fundamental life histories evolving through allocation‐dependent fecundity and survival

**DOI:** 10.1002/ece3.3730

**Published:** 2018-02-19

**Authors:** Jacob Johansson, Åke Brännström, Johan A. J. Metz, Ulf Dieckmann

**Affiliations:** ^1^ Evolution and Ecology Program International Institute for Applied Systems Analysis Laxenburg Austria; ^2^ Department of Biology Theoretical Population Ecology and Evolution Group Lund University Lund Sweden; ^3^ Department of Mathematics and Mathematical Statistics Umeå University Umeå Sweden; ^4^ Section of Theoretical Biology Institute of Biology and Mathematical Institute Leiden University Leiden The Netherlands; ^5^ Naturalis Biodiversity Center Leiden The Netherlands

**Keywords:** determinate growth, dynamic programming, indeterminate growth, marginal value theorem, reproductive allocation

## Abstract

An organism's life history is closely interlinked with its allocation of energy between growth and reproduction at different life stages. Theoretical models have established that diminishing returns from reproductive investment promote strategies with simultaneous investment into growth and reproduction (indeterminate growth) over strategies with distinct phases of growth and reproduction (determinate growth). We extend this traditional, binary classification by showing that allocation‐dependent fecundity and mortality rates allow for a large diversity of optimal allocation schedules. By analyzing a model of organisms that allocate energy between growth and reproduction, we find twelve types of optimal allocation schedules, differing qualitatively in how reproductive allocation increases with body mass. These twelve optimal allocation schedules include types with different combinations of continuous and discontinuous increase in reproduction allocation, in which phases of continuous increase can be decelerating or accelerating. We furthermore investigate how this variation influences growth curves and the expected maximum life span and body size. Our study thus reveals new links between eco‐physiological constraints and life‐history evolution and underscores how allocation‐dependent fitness components may underlie biological diversity.

## 1 INTRODUCTION

Simple life‐history models often predict that it is optimal to allocate all surplus energy to growth early in life, before switching to allocate all energy to reproduction. This allocation pattern is often referred to as a “bang‐bang control” and leads to determinate growth. Yet, simultaneous investment into growth and reproduction, leading to indeterminate growth, is common in nature. Much theoretical research on reproductive allocation has therefore investigated mechanisms and conditions, which can promote evolution of indeterminate growth, for example, stochastic environments (King & Roughgarden, 1982), diminishing returns of reproductive investments (Sibly, Calow, & Nichols, 1985; Taylor, Gourley, Lawrence, & Kaplan, 1974), or structural constraints (Kozłowski & Ziólko, 1988). This research (reviewed in Heino & Kaitala, 1999; Kozłowski, 1991; Perrin & Sibly, 1993) has established that simultaneous investment into growth and reproduction can be optimal, at least during some period of an organism's life. Less research has focused on investigating the shape and nature of the resulting mixed allocation patterns. Whereas bang‐bang control strategies can simply be characterized by the ages or sizes at which the switch from growth to reproduction occurs, it is less clear how to characterize and understand allocation schedules that cause reproductive investment to change gradually over a lifetime. Which shapes can we expect? What conditions favor different features in these shapes? How do particular optimal allocation schedules affect key life‐history features, such as growth curves, average life spans, or asymptotic body sizes?

Here, we pursue these questions for life‐history strategies that evolve under allocation‐dependent fecundity and mortality. We will specifically consider cases where fecundity and mortality rates increase with reproductive allocation, but at rates that may be either faster (henceforth referred to as accelerating) or slower (henceforth referred to as decelerating) than proportional. It is theoretically well established that simultaneous investment into growth and reproduction is favored when there are diminishing returns of reproductive investments. This occurs when fecundity increases at a decelerating rate with the fraction of surplus energy invested into reproduction or when mortality increases at an accelerating rate with the reproductive‐investment fraction (León, 1976; Sibly et al., 1985; Taylor et al., 1974).

There are many biological reasons why fecundity may depend nonlinearly on reproductive allocation. Competition between the offspring or between the gametes produced by an individual may cause diminishing returns from energy invested into reproduction. Eggs from a single mother may compete for resources, and sperm competition is common for both animals and plants (Andersson & Iwasa, 1996; Scharer, 2009). Structural constraints within an organism, for example, limited size of brood chambers in cladocerans (Perrin, Ruedi, & Saiah, 1987), can also lead to diminishing returns by impeding efficient use of surplus energy (cf. Kozłowski & Ziólko, 1988).

Accelerating returns from investment into reproduction may occur among plants in which increasing investments attract more seed‐dispersing or pollinating animals. For example, Sallabanks (1992) found that the proportion of seeds dispersed per plant increased with fruit abundance in hawthorn, *Crataegus monogyna*. By a similar token, Schaffer and Schaffer (1979) found accelerating returns produced by pollinators preferring the larger flowers in *Agavaceae*. Significant parts of the total energy invested into reproduction may not be channeled directly to offspring body mass but rather into organs or capacities which facilitate reproduction, for example, ovaries, inflorescences, or shells. Accelerating returns may then occur due to economies of scale, as the efficiency of such supportive features, in terms of cost per offspring, may increase with the size of the operation, for example, via reduced volume‐surface ratios or because the same facilities can be used many times. The finding by Greene and Johnson (1994) that trees with larger seeds invest a smaller proportion of energy into structures for protection and dispersal supports this idea (but see Lord & Westoby, 2012). Another example is learning in seabirds, whereby the probability that a breeding attempt is successful increases with the number of attempts, that is, with experience (Goodman, 1974).

Mortality is generally expected to increase with investment into reproduction (Calow, 1979; Calow & Woollhead, 1977; Sletvold & Ågren, 2015). Animals may, for example, be more vulnerable to predation when they are breeding or searching for partners, or be more susceptible to disease during the reproductive phase (Orton, 1929). As with fecundity, there are several reasons why also mortality may be a nonlinear function of reproductive allocation.

Mortality will increase at an accelerating rate with reproductive allocation if survival costs increase sharply when reproductive‐investment levels pass a threshold. For example, many systems of defence to diseases in plants require a minimal production of secondary tissue (Feeny, 1976; Fraenkel, 1959). If investment into growth is reduced as a result of surplus energy being channeled into reproduction, production of those tissues will decrease and lead to a sharply increased mortality rate. Further, as discussed by Bell (1980), growth of gonads in fish may have little effect on mortality as long as they are relatively small, but exert a strong negative effect if they exceed a critical proportion of the total body size and start compromising the functionality of organs necessary for survival. By contrast, mortality may increase at a decelerating rate with reproductive allocation when initial investments to reproduction are riskier for adults than additional investments. As an example, the mortality rate of zooplankton often increases during reproduction, as carrying eggs increases the chance to be detected by visual predators (e.g., Svensson, 1997). Because the risk of being detected is related to surface area rather than volume (e.g., Aksnes & Giske, 1993), one may expect that predation risk will increase at a less than proportional rate with the total number of eggs carried by a female. Other organisms that initially suffer high mortality risks are those that undertake long migratory journeys before reproducing, such as anadromous fish (cf. Bell, 1980; Gadgil & Bossert, 1970). For them, producing the first few eggs confers a high mortality risk, whereas continued egg production likely confers only little added risk.

Important qualitative insights into how the shape of optimal reproductive‐allocation schedules depends on fecundity and mortality rates have been obtained through mathematical investigations (León, 1976; Sibly et al., 1985; Taylor et al., 1974). However, although these studies are of a general nature, they do not give an overview of expected shapes of nonbang–bang reproductive‐allocation schedules. Numerical investigations in early pioneering studies (e.g., Gadgil & Bossert, 1970) are instructive in suggesting different possible shapes, but these investigations are limited to scenarios with a small number of age classes. Here, we attempt a systematic overview of the diversity of life‐history types that can arise from variation in the shape of fecundity and mortality functions, and of the consequences that different optimal reproductive schedules have for the growth, expected life span, and ultimate size of organisms. We shall focus on gradually increasing, accelerating, and decelerating fecundity and mortality functions, both because we believe these cases are biologically relevant, as described above, and for continuity with previous theory (e.g., Bell, 1980; Gadgil & Bossert, 1970; Sibly et al., 1985).

The remainder of this article is structured as follows. First, we introduce and describe a general life‐history model of ontogeny and procreation. Using dynamic programming, we determine optimal reproductive‐allocation schedules for each combination of generic fecundity and mortality regime. We systematically classify the emerging allocation schedules into twelve different classes depending on their characteristic shapes. We then study growth patterns and life spans associated with the twelve different types of optimal allocation schedules. We proceed by showing how our results can be understood at least in part by studying the marginal value of reproductive investment at different life stages. Finally, we synthesize our findings into a general, conceptual framework and discuss how they connect to empirical patterns.

## 2 MODEL

Our model builds on an established tradition in earlier studies of optimal allocation to reproduction (Cohen, 1971; Kozłowski & Ziólko, 1988; Perrin, Sibly, & Nichols, 1993; Sibly et al., 1985). An individual is assumed to produce energy at a mass‐dependent rate *E*. A fraction *u* of this energy is allocated to reproduction and the remaining fraction, 1 − *u*, is allocated to somatic growth. The mass *m* of an individual increases as

(1) $$dm/dt=\left(1-u(m)\right)E\left(m\right),\;m\left(0\right)=m_{\mathrm{birth}}.$$

The probability *P* of an individual surviving until age *t* decreases as

(2) dP/dt=−Pq(u),P(0)=1.

In the above equation, *q*(*u*) denotes the instantaneous mortality rate, which we assume to be an increasing function of the fraction *u* of available energy allocated to reproduction. This assumption is in line with Calow (1979) who argued that among several traditional alternatives, energy invested into reproduction as a proportion of the total energy income is the most suitable predictor of reproductive costs, such as mortality, and best reflects that these costs typically arise because reproduction competes for energy with other important physiological processes or activities. It also forms the basis for previous theory by, for example, Myers and Doyle (1983) and Sibly et al. (1985). The energy devoted to reproduction, *uE*, is converted into offspring biomass at a rate *b*(*uE*). Unlike the mortality rate *q*, which depends on the fraction invested into reproduction, the fecundity rate *b* is assumed to depend on the total amount of energy invested into reproduction.

**Figure 1:**
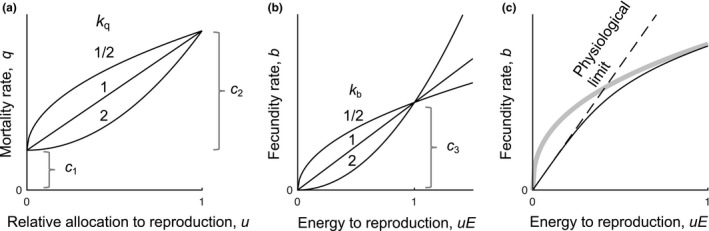
Fecundity and mortality rates as functions of reproductive investment. The panels illustrate how different parameters affect the shapes of these functions. The curvatures of the mortality functions in (a) depend on *k*_q_ and the curvatures of the fecundity functions in (b) depend on *k*_b_. (c) shows how the realized fecundity function (*b*, black line) is constructed as a smooth minimum of the potential fecundity ($\hat{b}$, gray line) and the physiological limit (dashed line)

In adherence to the existing tradition, we describe the dependence of vital rates on reproductive allocation using power functions (Figure 1). Specifically, we assume that the mortality rate is given by

(3) $$q\left(u\right)=c_{1}+c_{2}u^{k_{q}},$$

and the potential fecundity rate by

(4) $$\hat{b}\left(uE\right)=c_{3}{\left(uE\right)}^{k_{b}}.$$

As described below, we determine from the potential fecundity rate a realized fecundity rate *b* that determines the expected number of offspring of an individual. The two exponents *k*_q_ and *k*_b_ control whether the mortality rate and fecundity rate increase at an accelerating (*k*_*i*_ > 1), proportional (*k*_*i*_ = 1), or decelerating (*k*_*i*_ < 1) rate with *u* (see Figure 1).

The potential fecundity rate, Equation (4), has the unrealistic and undesirable feature that the slope becomes infinite at zero reproductive investment when *k*_b_ < 1. This means that a small increase in energy allocated to reproduction can convey an unrealistically large increase in the rate at which offspring are produced, potentially causing improbable evolutionary predictions. We avoid this problem by assuming a physiological limit such that whenever *k*_b_ < 1 the realized fecundity is always less than a factor *p* times the energy allocated (Figure 1c). Next, we construct a continuous and differentiable realized fecundity rate as a smooth minimum of the potential fecundity and the physiological limit by setting $b\left(uE\right)=(\hat{b}{\left({uE)}^{s}+{\left(puE\right)}^{s}\right)}^{1/s}$ for some value of *s* < 0, with increasingly negative values of *s* implying a closer approximation of the minimum.

Following the common assumption that biomass intake scales with mass according to a power law (e.g., Kozłowski & Wiegert, 1986; Reiss, 1989; Roff, 1992), the organism is assumed to acquire energy at a rate that depends on its current mass as

(5) $$E\left(m\right)=c_{4}m^{k_{e}}.$$

The expected lifetime reproduction of the individual is then given by

(6) $$R_{0}=\int_{0}^{\infty }P\left(t\right)\;b\left(u\left[m\left(t\right)\right]\;E\left[m\left(t\right)\right]\right)\;dt.$$

We aim to determine optimal mass‐dependent allocation schedules that maximize expected lifetime reproduction as given by Equation (6). This control problem is solved by applying the method of dynamic programming to a time‐discrete version of the ordinary differential equations (Equations [(1) and (2)]; see, e.g., Bertsekas, 1987 or Houston & McNamara, 1999 for an outline of the standard procedures used). Primarily, we vary the parameters *k*_q_, *k*_b_ and the physiological limit *p*, because they influence the curvatures of the fecundity and mortality functions. In line with previous studies (e.g., Charnov, 1993; Kozłowski & Uchmanski, 1987), we set the production exponent *k*_e_ to 3/4. Without loss of generality we reduce the model dimensionality by adjusting the timescale so that the baseline mortality rate, *c*_1_, equals 1. The remaining parameters are assigned values motivated by simplicity or chosen for illustrative purposes. We conduct a robustness check (see Appendix B) to clarify how variation of parameter settings and selected model assumptions influence optimal allocation schedules.

The optimal mass‐dependent allocation schedules are represented by the proportions u*(*m*) (where the asterisk denotes optimality) of energy invested into reproduction for any individual body mass *m*. We divide allocation schedules into different categories by considering the curvature of the function u*(*m*) for early and late life stages. To cover most of the biologically relevant life span, we calculate the trajectories of u* and *m* from *t* = 0 to a time‐point of low survival probability, *P* = 10^−6^. If *u* has not reached 1 within this time span, growth is considered to be indeterminate. We also estimate the maximum life span and maximum body size corresponding to the optimal schedules. Following a common practice in animal studies (e.g., Benedetti et al., 2008; Satoh et al., 2016), we define the maximum life span as the average age at death of the 10% most long‐lived individuals in a cohort. We then define maximum body size as the average final size of these individuals.

## 3 RESULTS

### 3.1 Types of optimal allocation schedules

**Figure 2:**
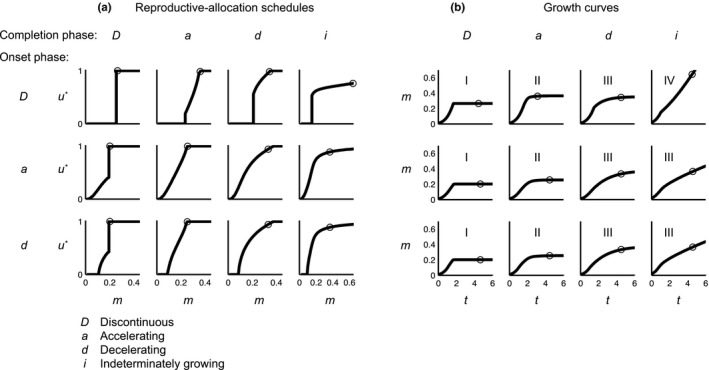
Different types of optimal reproductive‐allocation schedules (a) and corresponding growth curves (b). Letters indicate the shapes of the allocation schedules in the onset phase (rows) and in the completion phase (columns) of reproductive investment. For each allocation schedule shown in (a), the corresponding growth curve is shown in (b). The type of growth curve is indicated by the roman numbers I–IV in (b). The circles represent the mean age or mean size of the 10% oldest individuals. With some exceptions detailed below, we assume the following parameter settings: *k*_q_ = 0.95, 1.25, 3.5, and 10 in the columns from left to right, *k*_b_ = 1.02, 0.92, and 0.92 in the rows from top to bottom, *p* = 15, 15, and 1.2 in the rows from top to bottom, *c*_2_ = 0.04, *c*_1_ = *c*_3_ = *c*_4_ = 1, *m*_birth_ = 0.01, and *k*_e_ = 3/4. The exceptions are that for Da we set *c*_2_ = 1.15 and *k*_b_ = 1.1; for Di, we set *c*_2_ = 0.8; for aD, we set *c*_1_ = *c*_3_ = 2 and *k*_b_ = *k*_q_ = 0.95; and for dD, we set *c*_1_ = *c*_3_ = 2, *k*_b_ = *k*_q_ = 0.95, and *p* = 2.5

By exploring the salient parameter space, we identify twelve qualitatively different types of optimal allocation schedules u*(*m*). Figure 2a shows examples of each of the twelve types with parameter combinations selected for visual clarity. Reproductive allocation increases with mass either stepwise or gradually. As a consequence, the optimal allocation schedules consist of intervals with continuous increase and points with discontinuous increase (denoted D). Intervals of continuous increase can furthermore be divided into accelerating (denoted a) or decelerating (denoted d) functions of body mass. We also note that the optimal allocation schedules can have a different shape during the onset of reproduction (when u*(*m*) increases from zero) and during the completion phase (when u*(*m*) approaches 1 or an asymptotic value).

Four of the twelve types of allocation schedules (top row in Figure 2a) exhibit a discontinuous onset of reproduction, whereas the other eight are continuous in the beginning, increasing gradually from zero. In the completion phase, full reproductive allocation (*u* = 1) is approached discontinuously in three of the twelve types (leftmost column in Figure 2a). In the other nine types, the allocation curve intersects with *u* = 1 as a continuous curve that is either accelerating or decelerating (with the completion phase denoted a or d). In three types (rightmost column in Figure 2a), the allocation curve is decelerating without ever reaching full reproduction (*u* = 1), and this special case is categorized as indeterminate growth (with the completion phase denoted i).

Combinations of the three categories of shapes in the onset phase (D, a, or d) and the four categories of shapes in the completion phase (D, a, d, or i) constitute the twelve qualitatively different reproductive‐allocation schedules shown in Figure 2. In the following, we will use these onset‐ and completion‐phase shape categories to describe the different optimal mass‐dependent reproductive‐allocation schedules in abbreviated form. As an example, the string Dd refers to a reproductive‐allocation schedule with a discontinuous onset phase and a continuous, decelerating completion phase.

### 3.2 Growth patterns of the optimal types

Determined according to using Equation (1), the growth curves of the twelve numerically obtained optimal types described above are shown in (Figure 2b). The growth curves all have an accelerating phase in the beginning, since then investment into reproduction is low and almost all energy is used for growth. The growth curves differ more in the later stages. The types with a discontinuous completion phase stop growing suddenly (growth type I, first column in Figure 2b). The types with a continuous completion phase that reach maximal investment into reproduction, *u* = 1, before the survival probability falls below the stipulated threshold of *P* = 10^−6^ (growth type II, second column in Figure 2b), grow asymptotically toward a final mass. Growth curves for the types that do not reach *u* = 1 within this time frame are either decelerating (growth type III, third column in Figure 2b) or accelerating (growth type IV, fourth column in Figure 2b). In summary, the twelve qualitatively different types of reproductive‐allocation schedules correspond to four qualitatively different modes of growth late in life.

### 3.3 How optimal allocation schedules depend on fecundity and mortality curvature parameters

**Figure 3:**
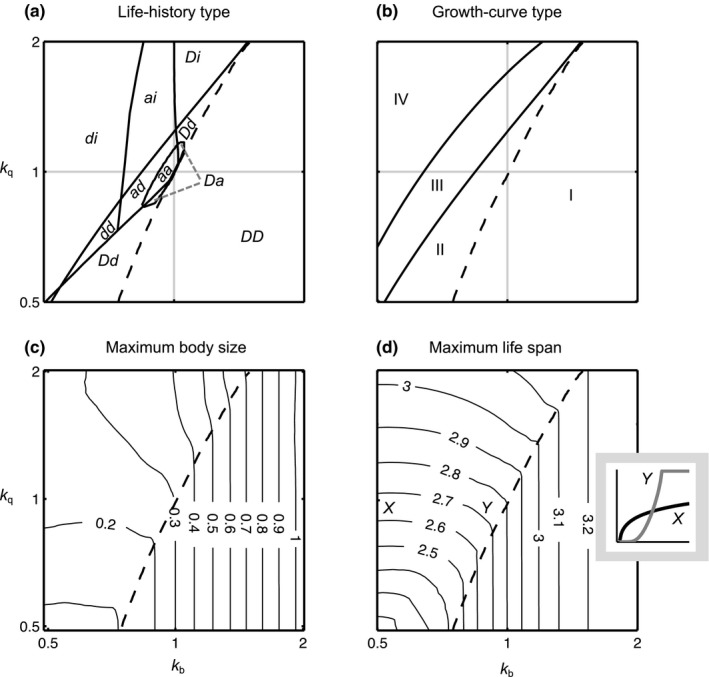
Overview of how the curvature parameters (*k*_b_, *k*_q_) of the fecundity and mortality functions affect the shape of the optimal reproductive‐allocation schedule and their properties. In (a) and (b), the lines indicate borders between different types. The types are classified as in Figure 2. (c) and (d) are contour plots. In (b–d) the dashed line indicates the border between bang‐bang control strategies (DD) and others. The gray lines in (a) and (b) correspond to *k*_b_ = 1 and *k*_q_ = 1, respectively. The inset in panel (d) shows the optimal allocation schedules u*(*m*) at the parameter values indicated by *X* and *Y* in the contour plot. Parameters: *c*_1_ = *c*_2_ = *c*_3_ = *c*_4_ = 1, *m*_birth_ = 0.01, *k*_e_ = 3/4, and *p* = 15

To find all twelve types of optimal allocation schedules in Figure 2a, it is necessary to vary more than two parameters at a time. It is therefore not straightforward to give a full overview of where in the parameter space different types are optimal. However, several types can be found by varying *k*_b_ and *k*_q_ while keeping the other parameters fixed (Figure 3a). We use this as a starting point for elucidating which conditions favor different types.

First, the two curvature parameters *k*_b_ and *k*_q_ determine whether the optimal allocation schedule is continuous, discontinuous, or a combination of the two. The four quadrants in Figure 3a correspond to combinations of accelerating (convex) and decelerating (concave) fecundity and mortality rates. In the top‐left quadrant, mortality is accelerating and fecundity is decelerating. These conditions are known to favor gradually increasing allocation to reproduction (see Sibly et al., 1985). Therefore, exclusively types with continuously increasing reproductive investment (aa, ad, ai, and di) are present there. In the opposite, lower‐right quadrant, none of these conditions are fulfilled, and thus, only the bang‐bang control strategy (DD) occurs there. In the top‐right and bottom‐left quadrants, one of the conditions for intermediate allocation is fulfilled, but not the other. It is only in these two quadrants that we find types with mixed discontinuous and continuous phases (Dd, Di, and Da). Heuristically, the combination of a curvature parameter that favors reproductive allocation of bang–bang type and a curvature parameter that favors graded allocation may yield types that are a mixture of the two. A more technical understanding can be gained by considering the marginal values of reproductive investment at different life stages, as we will detail in Section 3.5.

Second, the curvature parameters *k*_b_ and *k*_q_ determine whether optimal allocation schedules with gradually increasing reproductive allocation have accelerating or decelerating phases. This variation can be explained by the magnitude of the curvatures. Note, for example, that decreasing the curvature parameter *k*_b_ of the fecundity function and moving from (*k*_b_, *k*_q_) = (1, 1) to (*k*_b_, *k*_q_) = (0.5, 1) in Figure 3 induces a transition from an accelerating to a decelerating completion phase, and finally to indeterminate growth, which is a strongly decelerating completion phase. Similarly, increasing *k*_q_ in the three leftmost types in the middle row of Figure 2 leads from accelerating to increasingly decelerating completion phases.

In many cases, the shapes in the onset and completion phases do not coincide (all types except DD, aa, and dd). Consider, for example, the Dd and the dD types. Both exhibit continuously and discontinuously increasing allocation, but the Dd type has the discontinuous phase first and the dD type has the discontinuous phase last. The variation in the order by which different shape categories appear can be understood by observing how the curvatures of the fecundity and mortality functions vary with the level of investment. For example, the physiological‐limit parameter *p* affects the curvature of the fecundity function at low levels of investment (Figure 1c). Therefore, variation in *p* mainly affects the shape of the reproductive‐allocation schedule in its early phases. This explains why the early phases of the reproductive‐allocation schedules in the second and the third row in Figure 2a, which differ only in the parameter *p*, have different shapes (accelerating and decelerating, respectively). Next, consider the types aa, ad, da, and dd in Figure 2. These differ in the physiological‐limit parameter *p* and in the *k*_q_ parameter only. Variation in *p* causes a change between accelerating and decelerating curves in the early phases of investment, while variation in *k*_q_ causes the change between accelerating and decelerating curves in the later phases of investment (Figure 1a). Hence, by changing these parameters independently, all combinations aa, ad, da, and dd can be obtained.

In Appendix B we describe how variation in the other model parameters (*c*_1_, …, *c*_4_, *k*_e_, *P*, and *s*) qualitatively influence the shapes of the optimal schedules. We generally find that the diversity of optimal types is robust to moderate variation in these parameter values, but can be reduced if they take on very large or very small values. For example, several types in our scheme appear because of an interaction between the mortality and the fecundity functions. We might therefore get a lower diversity when effects from one of these dominate for example, if *c*_2_ is small compared to *c*_3_ such that variation in *u* will affect fecundity much more than mortality.

We also study how the optimal allocation schedules are affected by fixed costs of reproduction and size‐dependent mortality (see Appendix B). We find that fixed costs of reproduction, such that fecundity is zero until a minimum amount of energy is allocated to reproduction, have very similar effects on the shapes of optimal schedules as assuming accelerating fecundity. With regard to size‐dependent mortality, we find that if mortality is initially very high and decays only slowly with size we can get extreme and unrealistic outcomes where it is optimal to invest all energy to reproduction already from birth. However, if mortality drops relatively fast with size or is not too large overall, the effects on the shapes of the optimal allocation schedules, and thus also on overall diversity, should be relatively mild.

### 3.4 How growth‐curve types and life‐history attributes depend on fecundity and mortality curvature parameters

The allocation schedule determines the growth curve and, consequently, also the maximum life span and maximum body size of an organism. For example, the maximum body size increases as the allocation schedule becomes increasingly decelerating (e.g., second row in Figure 2b). In order to give a more systematic overview, we investigate how salient life‐history characteristics are affected by the shapes of optimal reproductive allocation under variation of the curvature parameters *k*_b_ and *k*_q_ (Figure 3b‐d). When moving toward the upper left corner (low *k*_b_ and high *k*_q_) in the parameter space, the types of growth (Figure 3b) change from ending abruptly (I) or asymptotically (II) to continued, indeterminate growth (III, IV). When comparing Figure 3a,b, it is also apparent that there is no complete overlap between the optimal types and the growth curves in the parameter space. Only the growth type that stops abruptly (I) exclusively corresponds to the DD type in Figure 3. However, with other parameter settings, this type of growth curve may also arise with an aD or a dD type (Figure 2b).

Variation in growth curves is associated with variation in maximum life span and maximum body size. As shown in Figure 3d, the bang‐bang control strategies (DD) and nonbang‐bang control strategies are affected differently by variation in the curvature parameters. For bang‐bang control strategies, the switch from none to full reproductive investment occurs at increasingly larger sizes when *k*_b_ is increased. The reason is that the stronger accelerating fecundity returns make it optimal to wait longer to reproduce. Hence, both maximum life span and maximum body size increase with *k*_b_. The bang‐bang control strategies are, however, not affected by *k*_q_, as they only take the values u* = 0 and u* = 1, and are therefore independent of *k*_q_ according to Equation (3). For nonbang‐bang control strategies, the effect of increasing *k*_b_ is that u* decreases at lower sizes and increases at higher sizes (moving from points *X* to *Y* in Figure 3d, inset), and as a consequence, individuals with the optimal allocation schedule experience lower mortality and grow faster when young, and experience higher mortality and grow slower when old. The net effect on maximum life span (maximum body size) depends on whether or not the increased survival (growth) in the earlier stages is compensated by the reduced survival (growth) in later stages.

### 3.5 The marginal values of reproductive investment

Qualitative insights into how fecundity and mortality functions affect the shape of the optimal allocation schedule can be gained by studying the returns of a short change in reproductive investment for the lifetime reproductive success of an individual. Following Metz, Staňková, and Johansson (2016) and in the spirit of the marginal‐value theorem (Charnov, 1976; see also Williams, 1966), we derive an expression for such fitness returns, denoted *r* for short (Appendix A). For the purposes of our arguments, *r* can be written as

(7) $$\begin{array}{cc} r(\tilde{u},t;u)= & \left[b\left(\tilde{u}E\left(m\right)\right)-b\left(uE\left(m\right)\right)\right]\left(t\right)-\left[\left(\tilde{u}-u\right)E\left(m\right)\right]\left(t\right)y_{1}\left(t,u\right)\\ & -\left[q\left(\tilde{u}\right)-q\left(u\right)\right]\left(t\right)y_{2}\left(t,u\right). \end{array}$$

Here, the first term gives the instantaneous return from reproduction if the reproductive allocation is increased from *u*(*t*) to $\tilde{u}$ at time *t* and the second and third term correspond to the future reduction in reproduction from the associated decrease in future sizes and decrease in survival probability, respectively. The expression allows us to compare the fitness return from changing the level of reproductive investment from *u* to $\tilde{u}$ at a given mass *m* of an individual. Here, $\tilde{u}$ can take on any value between 0 and 1, in contrast to the expression derived by Metz, Staňková, and Johansson (2016) that gives the marginal fitness returns, i.e. is, effects of small deviations from *u*. The factors *y*_1_(*t*, *u*) and *y*_2_(*t*, *u*) depend on the strategy *u* as a whole; explicit expressions are given in Appendix A. A reproductive‐allocation schedule u* is optimal if and only if the fitness return is maximized at $\tilde{u}=u^{*}\left(m\right)$ for any *m* ≥ *m*_birth_. Any deviation from the optimal strategy will thus either decrease lifetime reproduction or leave it unchanged, and the fitness return for the optimal strategy is always zero.

**Figure 4:**
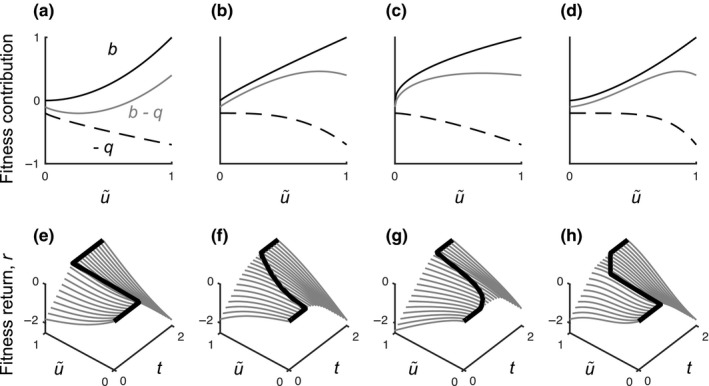
Illustration of how shapes of the optimal reproductive‐allocation schedules can be deduced using the fitness‐return approach. For an individual in a given state, the fitness return (*r* in Equation (7) of changing the allocation level from *u* to $\tilde{u}$ corresponds to a weighted mean (grey lines) of the functions for fecundity ($b(\tilde{u}E)$, continuous lines) and negative mortality ($-q(\tilde{u})$, dashed lines). Panels (a–d) show how the weighted mean qualitatively depends on the shape the fecundity and mortality functions. Panels (e–h) illustrate how the shape of the optimal allocation schedule (u*, thick solid lines) depends on the shape of the fitness‐return function ($r(\tilde{u},t;u^{*})$, grey lines) at different ages *t*. The fitness‐return functions in each panel (e–h) correspond to the weighted mean in the panels (a‐d) above

By analyzing the expression for fitness return, we see that the curvature of the fitness return *r* depends on the curvatures of the fecundity function *b* and the mortality function *q*. In fact, *r* has one term that is equal to $b(\tilde{u}E)$ and one term that is proportional to $-q(\tilde{u})$. The future fitness returns can be seen as a weighted mean of these two components. For example, when both *b* and −*q* are accelerating (i.e., have increasing slopes), their weighted mean, and thus *r*, will also be accelerating (Figure 4a). Likewise, when both *b* and −*q* are decelerating (i.e., have decreasing slopes), *r* will be decelerating too (Figure 4b,c). Finally, when *b* and −*q* have different curvatures, *r* may take on a sigmoidal shape (Figure 4d).

**Figure 5:**
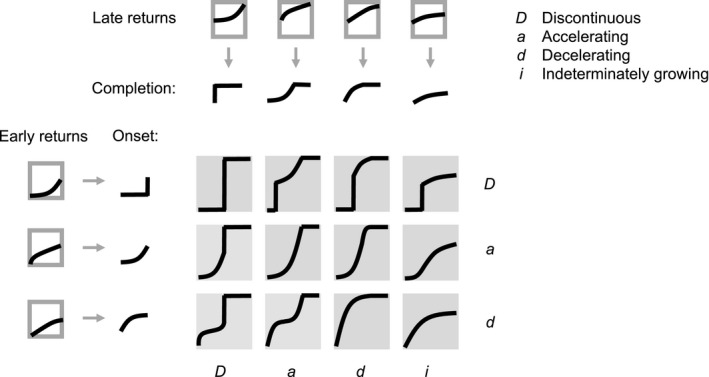
Conceptual map from allocation‐dependent fitness returns to optimal allocation schedules. The curves within the boxes framed in grey represent the shape of the fitness return (vertical axes) as a function of reproductive allocation *u* (horizontal axes) for early ages (left column) and late ages (top row), respectively. These shapes in turn map to shapes of the optimal reproductive‐allocation schedule, at the onset and completion of reproductive investment, respectively, as indicated by the grey arrows. The twelve types of optimal allocation schedules (boxes filled with grey) are then obtained as combinations of the three types of shapes at the onset phase and the four types of shapes at the completion phase. As in Figure 2a, optimal allocation schedules are shown as functions describing how the allocation level u* (vertical axes) depends on the of size *m* (horizontal axes)

Based on these observations, we can make qualitative predictions about the optimal allocation schedules when *b* and −*q* have the forms in Figure 4a–d. With an accelerating *r* (Figure 4a), there cannot be an interior maximum, so when its slope changes, changes in allocation must occur in the form of a sudden, discontinuous shift (Figure 4e). With a decelerating *r* (Figure 5b,c), there can be an interior maximum. As the slope of *r* changes in these cases, the maximum will increase gradually from 0 to 1, rendering a continuous allocation schedule (Figure 4f,g). Finally, with a sigmoidal *r* (Figure 4d), we may get a mixture of discontinuous and continuous allocation schedules (Figure 4h). Notice that in this example, the weighted mean is accelerating for low values of $\tilde{u}$ and decelerating for high values of $\tilde{u}$ (Figure 4d), which explains why the optimal allocation schedule first exhibits a discontinuous increase to then increasing continuously.

Whether a gradually increasing optimal allocation schedule will be accelerating or decelerating as a function of mass (e.g., types aa or dd) depends on more subtle properties of the fitness components. Consider the weighted mean of the fecundity and mortality in Figure 4b. Here, the birth rate *b* is nearly linear in $\tilde{u}$ and *q* is more curved for higher values of $\tilde{u}$. The weighted mean will therefore have most curvature at higher values of $\tilde{u}$. As the slope changes with age in this case, the maximum will increase relatively much at young ages and relatively little at older ages, thus giving rise to a decelerating shape. In Figure 4c, by contrast, *b* has higher curvature than *q*, and the weighted mean has highest curvature for low values of $\tilde{u}$. Therefore, the maximum will increase slowly at young ages and fast at old ages, resulting in an accelerating shape of the optimal allocation schedule. Note, however, that whether the accelerating function of time also will be an accelerating function of mass, in addition depends on the growth curves (which describe mass as a function of time). Thus, the relationship between early and late curvature of the weighted mean and accelerating and decelerating shape of u*(*m*) should be seen more as a tendency than as a strict rule.

### 3.6 Summary and synthesis

Three key observations from numerical investigations (Figures 2 and 3) and from studying the fitness returns of reproductive investments (Figure 4) can be used to summarize our results. First, an interval of a fitness‐return curve that is accelerating leads to an optimal reproductive‐allocation schedule with discontinuous increase (cf. Figure 4a,e), while decelerating intervals lead to an optimal reproductive‐allocation schedule with gradually increasing allocation (cf. Figure 4b,f). Second, the slope of a decelerating fitness‐return curve affects the shape of gradually increasing optimal reproductive‐allocation schedules, such that more deceleration causes a transition from an accelerating to a decelerating gradual increase (cf. Figure 4b,f with c,g). Third, the shape of the fitness‐return curve for low (high) levels of reproductive investment affects the shape of optimal reproductive‐allocation schedules at low (high) ages or masses (cf. Figure 4d,f).

We synthesize these relationships into the general classification scheme of optimal allocation schedules shown in Figure 5 (corresponding to the numerically obtained allocation schedules in Figure 2). Variation in fitness‐return curvatures in the early stages (small investment levels) leads to three different categories of shape in the onset phase (rows in Figure 5). The onset of reproduction may be discontinuous (D) or continuous depending on whether the fitness‐return curve at early stages is accelerating or decelerating. Depending on how strong the deceleration is, a continuous onset can be decelerating (d) or accelerating (a). Variation in fitness‐return curvatures in the later stages (high investment levels) leads to four different categories of shape in the completion phase (columns in Figure 5). Depending on whether the fitness‐return curve at these investment levels is accelerating or decelerating, full reproductive allocation may be approached discontinuously (D) or gradually by a continuous curve (a, d, or i). Depending on how strong the deceleration is, the continuous allocation curve may be accelerating (a), decelerating (d), or decelerating without ever reaching *u* = 1 (i).

Compared to the traditional classification of life histories into types with determinate growth and types with indeterminate growth, three types (DD, dD, and aD) clearly exhibit determinate growth and three types (Di, di, and ai) clearly exhibit indeterminate growth. For the remaining six types, however, it is a somewhat arbitrary judgment whether they could be said to exhibit determinate or indeterminate growth (cf. Figure 3a,b).

## 4 DISCUSSION

Our results show that variation in the shape of allocation‐dependent fecundity and mortality rates can give rise to a surprising diversity in optimal allocation schedules, hitherto not appreciated in the literature. Below we discuss how our findings are linked to previous theory. We also discuss how the results can be used to interpret empirical patterns in allocation and growth strategies and to understand how ecological and physiological constraints influence life‐history evolution.

### 4.1 Relation to other theoretical approaches

Our results extend the more qualitative results reported in earlier analytic studies (e.g., Sibly et al., 1985; Taylor et al., 1974). For example, Sibly et al. (1985) discussed which combinations of curvatures in fecundity and mortality rates render graded allocation schedules optimal, without going into further detail about the shapes of these schedules. By contrast, our results give concrete insights into what kind of diversity we can expect to arise from these mechanisms. We also show how this diversity can be understood by studying the fitness returns of reproductive investment (Equation (7), Figure 5, Appendix A) and thus shed light on how specific features of fecundity and mortality rates influence optimal allocation schedules.

The fitness return, which can be determined for any *u*, is in many ways similar to the fitness gradient in adaptive‐dynamics theory for the evolution of function‐valued traits (Dieckmann, Heino, & Parvinen, 2006; see also Metz et al., 2016). This connection provides an inroad to study allocation problems under the influence of environmental feedback (see also Parvinen, Dieckmann, & Heino, 2013). This is an interesting possibility for extensions of our present model, as optimization approaches inherently are directly applicable to a relatively narrow range of competitive scenarios, such as nursery competition (Metz, Mylius, & Diekmann, 2008). With an environmental feedback loop in place, we have to distinguish between primary parameters and parameters modified by the environmental feedback, like the full range of possible fertilities and that of fertilities in environments depleted by the corresponding equilibrium populations. If we allow maximal freedom in the primary parameters, up to natural physiological restrictions, the modified parameters can only be more restricted. Hence, incorporating an environmental feedback can only decrease the possible variation in outcomes of the optimization problem. We also note that the use of fitness returns together with an associated marginal‐value theorem to solve allocation problems methodologically connects our work to a large range of other problems in evolutionary theory, including mating behavior (Parker, 1974) and optimal foraging (Charnov, 1976).

While we have studied the influence of allocation‐dependent fecundity and mortality rates on optimal allocation schedules, similar types and similar diversity may result from other mechanisms as well. King and Roughgarden (1982) found, for example, that a discontinuous onset of reproduction followed by a continuous completion phase can be optimal for annual species when season lengths fluctuate stochastically. As another example, Janczur (2009) found that a qualitatively similar allocation schedule can be optimal for plants affected by herbivory, and furthermore, that the exact allocation level during the completion phase depended on the costs and efficiencies of chemical defense substances.

Theoretical studies have also predicted optimal reproductive‐allocation schedules with qualitatively different shapes compared to those we identify here. One example is given by McNamara, Houston, Barta, Scheuerlein, and Fromhage (2009), who investigated a model in which damage accumulated over an organism's lifetime make simultaneous allocation to growth and reproduction optimal. In contrast to our model, in which reproductive allocation always increases with age or remains constant, they showed that reproductive allocation could decrease at later stages of an organism's life when physiological conditions deteriorate. As another example, models taking into account seasonal variation in the environment predict that it may be optimal for perennial life histories to switch between phases of growth and reproduction every year (e.g., Kozłowski & Uchmanski, 1987) instead of simultaneously allocating to growth and reproduction, as we have studied here. Seasonally varying survival prospects for the offspring can further influence the optimal shape of such indeterminate growth patterns and affect whether it is optimal to invest into reproduction before or after growth within a single season (Ejsmond, Czarnołeski, Kapustka, & Kozłowski, 2010).

Because different alternative mechanisms may favor indeterminate growth, it would be of interest to know how our predictions regarding shapes of optimal allocation schedules and their diversity might be affected by alternative mechanisms, which also may explain indeterminate growth. Some insights into this question were provided in Klinkhamer, Kubo, and Iwasa (1997) who considered seasonal variation as well as allocation‐dependent fecundity and mortality functions (corresponding to *k*_b_ and *k*_q_ being nonzero) in a model of perennial plants. Note that other studies (e.g., Ejsmond et al., 2010; Kozłowski & Uchmanski, 1987) of optimal reproductive allocation in seasonal environments assume that fecundity is proportional to reproductive allocation (*k*_b_ = 1). In line with our conclusions, Klinkhamer et al. (1997) found that the optimal yearly allocation to reproduction increased gradually with age when there were diminishing returns from investment into fecundity. However, when mortality increased at a decelerating rate with reproductive allocation, in which case our model predicts bang‐bang control is optimal, they instead found that it was optimal to reproduce only in certain years, with nonreproductive years in between, a pattern comparable to masting. As noted by Klinkhamer et al. (1997), such patterns of intermittent reproduction in practice correspond to bang‐bang control if survival after the first reproductive year is very low. These comparisons show that some of our results can be carried over to more complex scenarios, but that additional mechanisms, e.g. associated with the presence of storage organs, can yield qualitatively different predictions.

Previous theoretical studies have mostly focused on studying whether or not, or under which conditions, a certain mechanism can give rise to indeterminate growth. By contrast, exploring the effects of variation in parameters that are structurally important for the shape of reproductive schedules, as we have done here, clarifies which potential a given mechanism has to generate diversity in life histories. An interesting avenue for future research is to compare which patterns of life‐history variation are generated by which alternative mechanisms. It would also be interesting to study more systematically interactions among different mechanisms that independently may favor indeterminate growth (cf. Klinkhamer et al., 1997).

### 4.2 Predictions about empirical patterns

Our analysis reveals links between ecological and physiological constraints on life‐history evolution, on the one hand, and shapes and characteristics of the expected reproductive‐allocation and growth strategies, on the other hand. In particular, our model bridges between the allocation patterns expected from evolution and the curvatures of vital rates that affect fitness returns. If these curvatures are known, the type of optimal allocation schedule can be predicted. Similarly, the shape of an optimal type can be used to infer aspects of the shape of the underlying fitness‐return function.

The usefulness of establishing these links depends on how well the curvatures of the fitness‐return functions and the features of the allocation patterns can be observed empirically. Empirical studies have identified reproductive‐allocation schedules, that can be related to those found in our study. For example, Wenk and Falster (2015) evaluated the existing empirical evidence for diversity of reproductive‐allocation schedules among perennial plants. Among the 32 species included in their review for which reproductive‐allocation schedules had been quantified or could be inferred, they identified six distinct types. Specifically, their types corresponded to DD, Dd, dd, two versions of di (depending on whether reproductive allocation approached an asymptote or continued to increase), and one type, not present in our model, characterized by a completion phase with declining reproductive allocation. As another example, Ware (1980) estimated how much surplus energy was allocated to either growth or reproduction in Atlantic populations of different fish species using allometric functions of size. The study reported that the functions describing energy devoted to reproduction (corresponding to *uE* in our model) for the different species had larger exponents than the functions describing surplus energy (corresponding to *E* in our study). If we assume that reproductive effort *u* in our study for each population corresponds to the ratio between the two functions, and because the difference between the estimated exponents in all cases were above 0 and below 1 (Table 3 in Ware, 1980), we can deduce that *u* should be a decelerating function of mass (corresponding to the dd or di types in our framework).

For some species, researchers have also put forward empirical evidence for why fecundity is a nonlinear function of energy devoted to reproduction and have used this to explain why a certain reproductive strategy might be optimal. For example, Schaffer and Schaffer (1979) related pollination‐driven accelerating returns of reproductive investments to the bang–bang strategy (DD) of *Yucca wipplei* and Miller, Tenhumberg & Louda (2008) related diminishing returns of reproduction owing to insect herbivory to a graded allocation pattern (corresponding to the dd or di types in our framework) in a species of cactus (*Opuntia imbricata*).

Estimating energy allocated to growth and reproduction during the lifetime of an organism can be complicated and expensive, which may explain why such observations are rare (cf. Myers & Doyle, 1983; Wenk & Falster, 2015). Estimating shapes of fecundity and mortality functions is perhaps even more challenging owing to the temporal decoupling between the allocation decision and final effect on lifetime reproduction. However, even if it may be hard to get a firm grip on these key features from data, they correlate with other patterns, that may be easier to observe. To start with, our model predicts connections between different types of allocation schedules and qualitatively different growth curves. Our model also predicts how the curvatures of mortality and fecundity functions relate to maximum life span and maximum body size (Figure 3c,d). Predictions from our model about variation in energy‐allocation patterns can therefore be connected to data on growth curves, body sizes, and age. For example, Myers and Doyle (1983) used a model similar to ours to reconstruct mortality curvatures from data on the growth and reproductive success on different fish species. As the curvatures of mortality and fecundity functions ultimately depend on ecological and physiological constraints on life‐history evolution, variation in these functions can be assumed to vary with factors, that influence the relevant constraints. It would, for example, be interesting to study whether life histories vary along gradients of sibling competition (cf. Stockley & Parker, 2002) or depend on reproductive constraints (cf. Shine, 1988) in line with our model predictions. In sum, even if some components of our model may be hard to validate, the multitude of connections between model predictions and accessible data make us believe that there are many ways the model predictions usefully generate empirically testable hypotheses about patterns of diversity in life histories.

One specific possibility for future research derives from our result that the properties of bang–bang types and types with continuous increase in reproductive allocation depend very differently on variation in curvature parameters. For example, with settings as in Figure 3c,d, there would be a strong positive correlation between maximum life span and maximum body size among randomly sampled bang–bang types, but not among nonbang–bang types. It would be interesting to investigate whether such patterns can be observed in empirical data (cf. Blueweiss et al., 1978; Hendriks, 2007) by grouping species according to life‐history type and seeing if correlations between life span and body size differ between these groups.

Our investigation here goes beyond the traditional perspective of dividing allocation patterns into those leading to either determinate or indeterminate growth. Our findings thereby offer a conceptual foundation for studying intermediate cases, enabling the systematic exploration of richer and more nuanced variation in life histories. Our results also provide links between ecological and physiological constraints and these life‐history types. By establishing a new and wider scope for testable predictions, we hope these results will inspire continued research into understanding the full variation of life histories encountered in nature.

## CONFLICT OF INTEREST

None declared.

## AUTHOR CONTRIBUTIONS

JJ, ÅB, and UD initiated the study and designed the model. JJ analyzed the model, conceived the synthetic framework, and wrote the first draft of the manuscript. JAJM provided the marginal‐value arguments and analyses of fitness returns. All authors discussed the results and implications and contributed substantially to revisions.

## Figures and Tables

**Figure B1:**
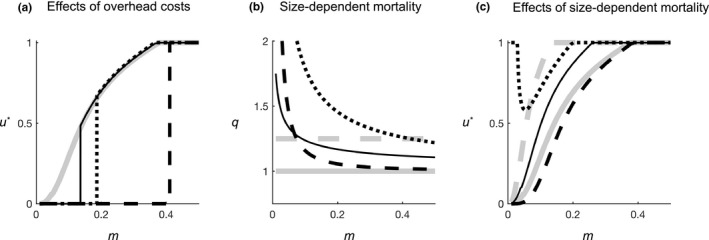
Robustness to fixed costs of reproduction (a) or size‐dependent mortality (b,c). We vary parameters in the modified functions for fecundity (a) or mortality (b,c) as described in the text; otherwise, parameter settings correspond to those of type ad in Figure 2a, which serves as a reference type. (a) Compared to the reference type (gray continuous line, *c*_5_ = 0), assuming fixed costs of reproduction (*c*_5_ > 0) causes the optimal reproductive‐allocation schedule to exhibit a discontinuous onset of reproduction (black continuous and dotted lines with *c*_5_ = 0.01 and 0.02, respectively) or be of bang–bang type (black dashed line, *c*_5_ = 0.1). (b,c) Compared to the reference type (gray continuous line, *c*_6_ = 0), size‐dependent mortality can displace the optimal allocation schedule to the left (black continuous line, *c*_6_ = 0.75, *k*_m_ = 0.5), to the right (black dashed line, *c*_6_ = 5, *k*_m_ = 1.5), or make it u‐shaped (black dotted line, *c*_6_ = 5, *k*_m_ = 0.8). The effects of increasing constant mortality by 25% with 25% compared to the baseline are shown for comparison as a gray dashed line (*c*_6_ = 0.25, *k*_m_ = 0). In (b) we set *u* = 0 to illustrate the effect of the size‐dependent mortality component only

## APPENDIX A Derivation of fitness‐return function

### A.1

Below we calculate the fitness returns according to Equation (7) for our optimization problem defined by Equations ((1), (2), (3), (4), (5), (6)). As in Metz et al. (2016), fitness returns $r(\tilde{u},t;u)$ denote the effects on the lifetime reproduction *R*_0_ of an individual that result from changing *u* to the constant value $\tilde{u}$ starting at *t* > 0 for a short duration of time, with the difference that we here consider changes in *u* that are not necessarily small. We assume that the control *u*, as a function of time, is piecewise continuously differentiable, which means that our analysis allows for controls of bang–bang type (DD) and other types with discontinuous increase in reproductive allocation. As the value of the control at finitely many points does not matter for the analysis, we assume that the time *t* > 0 does not correspond to a discontinuity of the control *u*.

We consider a change of the relative allocation to reproduction, *u*(τ), for τ ∊ [*t*, *t* + δ), say, to $\tilde{u}$, while leaving the rest of *u* unchanged. We denote by Δ_*m*_ the difference that this makes in mass *m*, and by Δ_*P*_ the difference that this makes in survival probability *P*. During the period when the perturbation is active, between ages *t* to *t* + δ (with *u* denoting the allocation function to which we make the change), we have

(8) $$\begin{array}{cc} & \frac{d\Delta_{m}}{d\tau }=\frac{d\tilde{m}}{d\tau }-\frac{dm}{d\tau }=\left(1-\tilde{u}\right)E\left(m+\Delta_{m}\right)-\left(1-u\right)E\left(m\right)=-\left(\tilde{u}-u\right)E\left(m\right)+O\left(\delta \right),\;\Delta_{m}\left(t\right)=0,\\ & \frac{d\Delta_{P}}{d\tau }=\frac{d\tilde{P}}{d\tau }-\frac{dP}{d\tau }=-q\left(\tilde{u}\right)\left(P+\Delta_{P}\right)+q\left(u\right)P=-\left(q\left(\tilde{u}\right)-q\left(u\right)\right)P+O\left(\delta \right),\;\Delta_{P}\left(t\right)=0. \end{array}$$

The first equation holds as *m* is differentiable at *t* and therefore *m*(τ) = *m*(*t*) + O(δ) for τ ∊ [*t*, *t* + δ). Now, as a direct consequence of Equation (A1), we have

(9) $$\begin{array}{cc} & \Delta_{m}\left(t+\delta \right)=-E\left(m(t)\right)\left(\tilde{u}-u\left(t\right)\right)\delta +O\;\;\left(\delta^{2}\right),\\ & \Delta_{P}\left(t+\delta \right)=-\left(q\left(\tilde{u}\right)-q\left(u(t)\right)\right)P\left(t\right)\delta +O\;\;\left(\delta^{2}\right). \end{array}$$

We use these intermediate results to derive an expression for the fitness return, proceeding in four steps. First, we note that the immediate fitness gain from this strategy change for an individual that already has survived until time *t* is given by

(A3) $$\delta \;\left[b\;\left(\tilde{u}E\left(m\right)\right)\;-\;b\;\left(uE\left(m\right)\right)\right]\left(t\right)+O\left(\delta^{2}\right).$$

Second, by linearizing the integrand in the expression for lifetime reproduction, i.e., *P*(*t*)*b*(*u*(*t*)*E*(*m*(*t*))) in Equation (6), we approximate the future fitness loss resulting from this change in strategy as

(A4) $$-\frac{1}{P(t)}\int_{t}^{\infty }\left[\Delta_{P}b\left(uE(m)\right)+\Delta_{m}Pb^{\prime }\left(uE(m)\right)uE^{\prime }\left(m\right)\right]\left(\tau \right)d\tau .$$

Note that we here divide with *P*(*t*) because we again consider the fitness effects for an individual that has survived until time *t*.

Third, we determine linear approximations of Δ_*P*_(τ) and Δ_*m*_(τ) for the time period after the perturbation, i.e. for τ > *t* + δ. To this end, we use that the differential equation for *P*(τ)/*P*(*t* + δ) with respect to τ is the same as that for Δ_*P*_(τ), except for a difference in initial conditions. We define $\hat{P}\left(\tau ;t\right)$ and $\hat{m}\left(\tau ;t\right)$ by

(A5) $$\frac{d\hat{P}}{d\tau }=-q\left(u\right)\hat{P},\;\hat{P}\left(t;t\right)=1,\;\frac{d\hat{m}}{d\tau }=\left(1-u\right)E^{\prime }\left(m\right)\hat{m},\;\hat{m}\left(t;t\right)=1.$$

$where\hat{P}$ and $\hat{m}$ are functions of both τ and *t*. However, we shall from now on hide the latter argument. Using Equations (A2) and (A5), we get $\Delta_{P}\left(\tau \right)=\Delta_{P}\left(t+\delta \right)\hat{P}\left(\tau \right)=-P\left(t\right)\left(q\left(\tilde{u}\right)-q\left(u\left(t\right)\right)\right)\delta \hat{P}\left(\tau \right)+O\left(\delta^{2}\right)$ and $\Delta_{m}\left(\tau \right)=\Delta_{m}\left(t+\delta \right)\hat{m}\left(\tau \right)=-E\left(m\left(t\right)\right)\left(\tilde{u}-u\left(t\right)\right)\delta \hat{m}\left(\tau \right)+O\left(\delta^{2}\right)$. We also note that $P\left(\tau \right)=P\left(t\right)\hat{P}\left(\tau \right)+O\left(\delta \right)$ and we will use this below to express all survival rates in terms of $\hat{P}\left(\tau \right)$.

Fourth we express the fitness return *r* as the difference between the immediate fitness gain (Equation [A3]) and the future fitness loss (Equation [A4] with Δ_*P*_(τ) and Δ_*m*_(τ) replaced by the approximations above), divided by δ. We will also ignore higher‐order terms. Thus, we find

(A6) $$\begin{array}{cc} r(\tilde{u},t;u) & =\left[b\left(\tilde{u}E\left(m\right)\right)-b\left(uE\left(m\right)\right)\right]\left(t\right)\\ & \;-\int_{t}^{\infty }\hat{P}\left(\tau \right)\left[E\left(m\left(t\right)\right)\left(\tilde{u}-u\left(t\right)\right)b^{\prime }\left(uE(m)\right)uE^{\prime }\left(m\right)\hat{m}+\left(q\left(\tilde{u}\right)-q\left(u\left(t\right)\right)\right)b\left(uE(m)\right)\right]\left(\tau \right)d\tau \\ & =\left[b\left(\tilde{u}E\left(m\right)\right)-b\left(uE\left(m\right)\right)\right]\left(t\right)-\left(\tilde{u}-u\left(t\right)\right)E\left(m\left(t\right)\right)\int_{t}^{\infty }\left[\hat{P}b^{\prime }\left(uE(m)\right)uE^{\prime }\left(m\right)\hat{m}\right]\left(\tau \right)d\tau \\ & \;-\left(q\left(\tilde{u}\right)-q\left(u\left(t\right)\right)\right)\int_{t}^{\infty }\left[\hat{P}b\left(uE(m)\right)\right]\left(\tau \right)d\tau . \end{array}$$

In our comparison (Figure 4), we focus on how the fitness returns through the different vital rates affect the form of the optimal allocation *u**. In order to facilitate reading, we rename the first and second integral in Equation (A6), which both are independent of $\tilde{u}$, as *y*_1_(*t*, *u**) and *y*_2_(*t*, *u**) in Equation (7). At $\tilde{u}\left(t\right)=u^{*}\left(t\right)$, the total return *r*(*t*) is 0 when 0 < *u**(*t*) < 1, nonpositive when *u**(*t*) = 0, and non‐negative when *u**(*t*) = 1. As shown by Metz et al. (2016), it may be noted that for the optimal allocation strategy, the functions *y*_1_(*t*, *u**) and *y*_2_(*t*, *u**) correspond to the so‐called costates from Pontryagin's maximum principle from optimal control theory (Intrilligator, 1971; Pontryagin, 1962). As in the present article we aim at providing biological insight, we have opted for a biologically inspired argument in terms of fitness returns and a marginal‐value consideration, instead of falling back on the traditions of mathematical control theory.

## APPENDIX B Robustness and sensitivity of numerical results

### B.1

In order to evaluate the robustness of our approach, we study here how the shapes of the optimal allocation schedules are affected by (1) variation in model parameters, (2) explicit fixed costs of reproduction, and (3) size‐dependent mortality. We mainly consider the effects on the diversity of optimal types, which is main focus of this study.

#### Effects of variation in model parameters

We identify two major effects of varying the parameters *c*_1_, …, *c*_4_ and *k*_e_. First, variation in these parameters can make it optimal to switch to reproduction earlier or later in life. Specifically, earlier investments to reproduction are optimal when mortality increases (increased *c*_1_ or *c*_2_) or when fecundity or growth efficiency decreases (increased *c*_3_ or *c*_4_), all in line with general expectations. These changes do not necessarily affect the shapes of the optimal allocation schedules, but in extreme cases, it may become optimal not to grow at all and invest all surplus energy to reproduction from birth onward (e.g., for very high *c*_1_). Close to such unrealistic conditions, the model will exhibit a lower diversity of types. Increasing *k*_e_ may either increase or decrease fecundity returns depending on body mass (note, e.g., that *E* in Equation (5) will decrease with *k*_e_ if *m* < 1, but increase otherwise), such that *u**(*m*) decreases (increases) for small (large) body masses, potentially resulting in a rather flat *u**(*m*). If the latter effect is strong, the potential for diversity to arise through other mechanisms naturally decreases.

Second, variation in the parameters *c*_2_, …, *c*_4_ influences whether the shape of the optimal allocation schedule is mainly determined by allocation‐dependent mortality or by allocation‐dependent fecundity. If *c*_2_ is large compared to *c*_3_ and *c*_4_, the shape of the optimal type will mainly depend on the mortality curvature *k*_q_, and in the opposite situation, the shape will mainly depend on the fecundity curvature *k*_b_. Because several types in our scheme (e.g., dD, aD, and ad) appear because of an interaction between the mortality and the fecundity function, we get low diversity when effects from one of these dominate over effects from the other.

The overarching result from these observations is that the diversity of optimal types may be constrained if these general model parameters take on relatively large or small values. Extreme values of the parameters *p* and *s*, which are introduced by us to control the physiological limit (Figure 1c), may also reduce diversity or have undesired effects. As noted in the Results section, high values of the parameter *p* favor types with accelerating (a) onset of reproduction and lower values favor types with decelerating (d) onset. In addition, if *p* is set to a very low value, fecundity will be a linear function (*b*(*uE*) = *puE*), which causes the onset to be discontinuous (D). It follows that variation in the parameter *p* may reduce the diversity of optimal types if it is made too small or too large. The parameter *s* controls how close the smooth minimum function fits the standard minimum function. With very large negative values of *s*, the fecundity function will have a sharp transition from a linear to a nonlinear section, which causes numerical instability when determining the optimal allocation schedule. When *s* has values close to zero, on the other hand, the smooth minimum function starts deviating a lot from the standard minimum function and is hence not a meaningful approximation anymore. We also examine the survival probability (*P* = 10^−6^) according to which growth is classified as indeterminate. Specifically, we considered effects on the line in Figure 3a that separates indeterminate from determinate growth. Decreasing P displaces this line upwards and to the left, but only relatively little, as many optimal allocation schedules in the top‐left corner seem never to reach full reproduction (*u** has an asymptote below 1). Increasing P, however, can move this line arbitrarily far down and right, which is expected, as organism then have less time to reach full reproductive allocation (*u* = 1) before their survival probability falls below the critical level.

#### Effects of fixed costs reproduction

While in this study we assume accelerating fecundity functions (*k*_b_ > 1), which may represent a need for initial investments to be made before energy can be diverted into offspring directly, we also explore an alternative way to model such situations Following Charnov (1979), we assume a fixed cost *c*_5_ for reproduction by specifying the energy devoted to offspring as *f*(*uE*) = max (0, *uE* − *c*_5_) and assuming the fecundity $b\left(uE\right)=c_{3}f{\left(uE\right)}^{k_{b}}$. As illustrated by Figure B1a, where we use the type ad as a baseline, these costs cause the onset of reproduction to be discontinuous. When the fixed cost is increased, reproduction starts later and the discontinuous jump becomes larger, eventually making a bang–bang strategy optimal. The same occurs when applying fixed costs to the other types with continuous onset in Figure 2a. Fixed costs modeled in this way thus have effects similar to increasing *k*_b_ in our model. Notice, for example, that when moving toward the right in Figure 3a, the optimal types typically first develop a discontinuous onset of reproduction (Da or Dd) and then turn into bang–bang strategies (DD).

#### Effects of size‐dependent mortality

As a further robustness test, we explore the effects of assuming size‐dependent mortality, given its common occurrence in nature (e.g., Paine, 1976). To this end, we add a size‐dependent term to Equation (1), representing increased mortality for juveniles. Specifically, we assume $q\left(u,\;m\right)=c_{1}+c_{2}u^{k_{q}}+c_{6}m^{k_{m}}$ (cf. Sibly et al., 1985). We explore the effects of varying *c*_6_ and *k*_m_ on the optimal allocation schedules in Figure 2a. As in the previous section, we use the type ad from Figure 2a for reference. The results are nevertheless representative for the remaining types there as well.

In three scenarios with size‐dependent mortality, we observe relatively small changes in the shapes of the optimal allocation schedules. Firstly when mortality is initially moderate and then declines relatively slowly with size (black continuous line in Figure B1b), the optimal allocation schedule is displaced to the left, that is, reproduction occurs earlier (black continuous line in Figure B1c). This can be interpreted as an effect of increased average mortality, similar to the effect of an increased size‐independent mortality (gray dashed lines in Figure B1b,c). Secondly, if mortality is initially high and then drops relatively fast with size (black dashed line in Figure B1b), the optimal allocation schedule is displaced to the right, that is, reproduction is delayed (black dashed lines in Figure B1c). This occurs because by growing as fast as possible when young, individuals can avoid the high juvenile mortality. Thirdly, when *k*_m_ is sufficiently large, the optimal allocation schedule remains virtually unchanged (i.e., it is nearly identical to the gray continuous line in Figure B1c and thus not shown). This occurs because the size‐dependent component of mortality quickly vanishes as the individual grows, and it thus remains optimal to grow fast (low *u**) at small sizes.

Although the size‐dependent mortality functions discussed so far (e.g., the black continuous and dashed lines in Figure B1b) are qualitatively different from the constant mortality function in the baseline case (gray continuous line in Figure B1b), their effects on the optimal allocation schedules are comparable to the effects of relatively minor variation of the parameters of the original model. Note, for example, that increasing the (size‐independent) mortality of the original model by 25% (i.e., moving from the gray continuous to the gray dashed line in Figure B1b) has a larger effect on the size at which full reproduction is reached (*u** = 1) than any of the three scenarios discussed so far. Also, none of these scenarios causes a change in the shape of the optimal allocation schedules according to our classification system (i.e., for the type ad in Figure B1b and the remaining 11 types in Figure 2b in the main text).

In two other scenarios, we observe more drastic effects of including size‐dependent mortality in our model. Firstly, the optimal allocation schedule can become u‐shaped (dotted line in Figure B1b,c). This occurs when mortality is initially very high and drops relatively slowly, such that low survival prospects initially make it optimal to invest in reproduction from birth onward and it pays off to grow only starting at larger sizes. Such a strategy is, however, not biologically feasible, as growth would stop immediately upon birth. Secondly the extreme and unrealistic strategy of devoting all energy to reproduction from birth onward (*u**(*m*) = 1 for all *m*), is optimal when mortality is high for all sizes (when *c*_6_ is large and *k*_m_ small).

In sum, adding a mortality component, that decays with size only slowly, can lead to extreme situations in which it is optimal to invest all energy into reproduction already from birth. However, if mortality drops relatively fast with size or is not too large overall, we expect relatively mild effects on the shapes of the optimal allocation schedules, and thus also on overall life‐history diversity.
